# Organ crosstalk and dysfunction in Sepsis: harnessing emerging biotechnologies for future breakthroughs

**DOI:** 10.1186/s13613-024-01398-9

**Published:** 2024-10-23

**Authors:** Alexandre Pierre, Steve Lancel, Sebastien Preau

**Affiliations:** 1 Univ. Lille, CHU Lille, Institut Pasteur de Lille, U1167 - RID-AGE - Facteurs de risque et déterminants moléculaires des maladies liées au vieillissement, Inserm, Lille, F-59000 France; 2 Division of Intensive Care, Hôpital Roger Salengro, CHU de Lille, Lille, F-59000 France

## Text

As Dr. Borges and Dr. Bento pointed out in a recent issue of *Annals of Intensive Care* [1], the phenomenon of organ crosstalk in sepsis represents a crucial area of investigation, elucidating the intricate interactions between organs that drive the progression of multi-organ failure. Sepsis triggers a cascade of immune and metabolic responses, orchestrated by complex communication between different organ systems. This process involves multiple networks of long-distance interactions, such as the heart-lung-kidney axis and the gut-liver-brain connections, mediated by factors such as cytokines, hormones, miRNAs, extracellular vesicles and metabolites, which exert autocrine, paracrine and endocrine effects. A deeper understanding of these multifaceted mechanisms is essential for advancing strategies to monitor organ dysfunction and for developing targeted therapeutic approaches in sepsis.

Nevertheless, to date, most studies on sepsis primarily focused on individual organs or circulatory changes to test therapeutic hypotheses. This narrow focus may, in part, explain some of the failure of translational research in recent decades. The inherent complexity of sepsis requires a broader perspective, considering sepsis-induced changes at the whole-body level across multiple cells and tissues. Now, emerging biotechnologies offer significant potential for advancing research and patient care, and can be applied to the context of understanding organ crosstalk and dysfunction in sepsis.

### Profiling organ crosstalk with integrated multi-omics

Omics refers to a group of disciplines aimed at characterizing the complete set of molecules within a tissue using high-throughput, large-scale data acquisition methods, such as transcriptomics for RNA and metabolomics for metabolites. Without any prior hypotheses, these approaches provide high-resolution, data-rich insights into the structure and function of the tissue. (i) Recent developments now make it possible to capture the molecular information of cells and tissues molecules while maintaining their spatial location, known as spatial omics [2]. It provides new insights in the relationships and signaling pathways between different cells and tissues, uncovering regional heterogeneity. This approach could also facilitate the understanding of the body’s compartmentalised response to sepsis. For example, Takahama et al. recently investigated whole-body spatial transcriptomics to map dynamic gene expression changes across multiple organs, unveiling how organ dysfunction is driven by cytokine-induced signaling, providing a clearer picture of interorgan communication during sepsis [3]. (ii) Single-cell omics, such as single-cell RNA sequencing, is a powerful approach that identifies molecules at the resolution of individual cells, enabling detailed profiles of cellular heterogeneity within organs, according to cell type [2]. This approach offers a more comprehensive insight into the mechanisms of intercellular communication. The application of spatial and single-cell omics identifies overlapping metabolic pathways that are affected throughout the body, as well as those that are unique and specific to different cells and tissues. (iii) Multi-omics integration with new computational modelling strategies allows to integrate and resume the large omics datasets of different nature (from the DNA to the metabolite scale) and to interpret multiple biological pathways modifications into comprehensive information [2]. Machine learning helps to integrate vast datasets from multi-omics and imaging studies, and to identify patterns that would be impossible to detect using “traditional” analytical methods [2]. Overall, multi-omics approaches will undoubtedly aid in unravelling the intricate interactions between organs during sepsis, while advancing the discovery of biomarkers and personalized therapies.

### Modelling organ crosstalk with bio-engineering approaches

Beyond the “traditional” experimental models, such as sepsis murine models, bioengineering could help to better understand inter-organ dialogues with innovative experimental models. Multi-organ-on-chip, or body-on-a-chip, is a compartmentalized microfluidic device that replicates the structure and functions of human organs by isolating different cell types and processes into distinct microenvironments to simulate physiological behaviours [4]. Channels replicate the inter-organ circulation and allow the control of interactions between compartments by precisely regulating the flow of fluids and signals between different microenvironments, mimicking the dynamic exchanges that occur in real organs. For instance, this platform enables the detailed analysis of immune cells compartmentalization. By using a human multi-organ system, Sasserath et al. highlighted the specific characteristics of the localized inflammatory responses in tissues damaged by recirculating immune cells, distinguishing these from the systemic inflammatory responses [5]. In the context of sepsis research, it could be critical for elucidating how cells and tissues communicate with each other and for testing how targeted therapies affect multiple organs simultaneously.

## Concluding comments

An interdisciplinary approach, combining researchers from immunology, metabolism, bioinformatics, and bioengineering, along with ICU clinicians, aims to develop new tools to monitor organ crosstalk in sepsis. Implementing these new tools will enable the integration of cellular and molecular signals from each tissue, giving an “extensive microscopic description” at the systemic level that helps to address the ‘challenges of causal inference and directionality’ in sepsis.

Taken together, emerging biotechnologies are creating new paradigms in medicine and offers the possibility of interpreting the systemic message regarding the molecular and cellular context. Further research should leverage these technologies to deepen our understanding of organ crosstalk during sepsis, ultimately advancing the fields of diagnostics, therapeutics and personalized medicine.

## Data Availability

Not applicable.
